# Role for Rab10 in Methamphetamine-Induced Behavior

**DOI:** 10.1371/journal.pone.0136167

**Published:** 2015-08-20

**Authors:** Scott M. Vanderwerf, David C. Buck, Phillip A. Wilmarth, Leila M. Sears, Larry L. David, David B. Morton, Kim A. Neve

**Affiliations:** 1 Department of Behavioral Neuroscience, Oregon Health & Science University, Portland, Oregon, United States of America; 2 Department of Integrative Biosciences, Oregon Health & Science University, Portland, Oregon, United States of America; 3 Department of Biochemistry and Molecular Biology, Oregon Health & Science University, Portland, Oregon, United States of America; 4 Research Service, VA Portland Health Care System, Portland, Oregon, United States of America; Karolinska Institutet, SWEDEN

## Abstract

Lipid rafts are specialized, cholesterol-rich membrane compartments that help to organize transmembrane signaling by restricting or promoting interactions with subsets of the cellular proteome. The hypothesis driving this study was that identifying proteins whose relative abundance in rafts is altered by the abused psychostimulant methamphetamine would contribute to fully describing the pathways involved in acute and chronic effects of the drug. Using a detergent-free method for preparing rafts from rat brain striatal membranes, we identified density gradient fractions enriched in the raft protein flotillin but deficient in calnexin and the transferrin receptor, markers of non-raft membranes. Dopamine D1- and D2-like receptor binding activity was highly enriched in the raft fractions, but pretreating rats with methamphetamine (2 mg/kg) once or repeatedly for 11 days did not alter the distribution of the receptors. LC-MS analysis of the protein composition of raft fractions from rats treated once with methamphetamine or saline identified methamphetamine-induced changes in the relative abundance of 23 raft proteins, including the monomeric GTP-binding protein Rab10, whose abundance in rafts was decreased 2.1-fold by acute methamphetamine treatment. Decreased raft localization was associated with a selective decrease in the abundance of Rab10 in a membrane fraction that includes synaptic vesicles and endosomes. Inhibiting Rab10 activity by pan-neuronal expression of a dominant-negative Rab10 mutant in *Drosophila melanogaster* decreased methamphetamine-induced activity and mortality and decreased caffeine-stimulated activity but not mortality, whereas inhibiting Rab10 activity selectively in cholinergic neurons had no effect. These results suggest that activation and redistribution of Rab10 is critical for some of the behavioral effects of psychostimulants.

## Introduction

Methamphetamine abuse is a serious international public health problem, with the use of methamphetamine and other amphetamine-type stimulants exceeding the use of opiates or cocaine [1]. Long-term use of methamphetamine can lead to addiction, paranoia, agitation, psychosis, deficits in attention and memory, and motor dysfunction, and its discontinuation is often accompanied by a withdrawal syndrome [2]. The best-characterized effect of methamphetamine is stimulation of monoamine release mediated by methamphetamine-induced inhibition of the monoamine transporters and disruption of vesicular storage of monoamines [3,4]. Of the monoamine neurotransmitters, dopamine is particularly important for the reinforcing and locomotor-activating properties of psychostimulant drugs including methamphetamine [5,6]. More recently, the trace amine associated receptor TAAR1 has been identified as another likely site of action of methamphetamine that opposes its transporter-mediated dopamine-releasing effect [7–9]. Although the effects of methamphetamine on dopamine systems have been studied extensively, there is no pharmacotherapy currently available to treat methamphetamine abuse or addiction, and better insight into the molecular effects of methamphetamine is clearly needed.

To find new targets for modulating the effects of methamphetamine we used proteomics analysis to identify proteins whose abundance in rat neostriatal lipid rafts was altered by methamphetamine. Because lipid raft domains act as organizers of signal transduction pathways by restricting or promoting interactions between subsets of the cellular proteome, we hypothesized that protein expression differences would reveal novel mediators of the effects of methamphetamine. Many G protein-coupled receptors and other membrane proteins involved in signal transduction reside in raft domains and move in and out of these domains according to their activation state [10]. We reasoned that methamphetamine-induced changes in the raft proteome would be an indication of altered protein activity, and that the mechanisms responsible for such changes, such as altered protein-protein interactions or membrane trafficking, might play key roles in methamphetamine-induced behavior.

As reported herein, a number of proteins do move into or out of raft domains upon methamphetamine stimulation. One protein whose abundance in raft fractions was decreased by acute methamphetamine treatment was Rab10, a monomeric GTP-binding protein that functions as a regulator of intracellular membrane trafficking. Subcellular fractionation indicated that the decrease was in the vesicular/endosomal membrane compartment. We expressed a dominant-negative mutant of Rab10 (DN-Rab10) in neurons of *Drosophila melanogaster* and monitored their behavior after receiving methamphetamine in their diet. Rab10 inactivation in neurons of flies resulted in (a) reduced methamphetamine-dependent locomotion and (b) resistance to the lethality observed in flies fed a high concentration of methamphetamine. Rab10 inactivation inhibited caffeine-induced activity but not lethality. This work suggests that redistribution of Rab10 is critical for some of the behavioral effects of methamphetamine and other psychostimulants.

## Materials and Methods

### Antibodies and Reagents

A rabbit polyclonal antibody that recognize Rab10 (product #8906) was purchased from Sigma-Aldrich (St. Louis, MO, USA), mouse anti-flotillin-1 (product #610820) was supplied from BD Biosciences (San Jose, CA, USA), rabbit anti-calnexin (product #SPA-865) was from Assay Designs (Farmingdale, NY, USA), and mouse anti-transferrin receptor (product #13–6800) was purchased from Life Technologies (Grand Island, NY, USA). Cholesterol concentrations were measured using the cholesterol/cholesteryl ester quantitation kit (product #428901) from Calbiochem (EMD Millipore, Billerica, MA, USA). Methamphetamine was provided by the National Institute on Drug Abuse Drug Supply Program.

### Methamphetamine Treatment and Tissue Preparation

Male Sprague-Dawley rats were treated with methamphetamine (2 mg/kg, i.p.) or saline either one time only or once daily for 11 days and euthanized by CO_2_ asphyxiation followed by decapitation 30 min after the final injection. Striata were rapidly dissected on an ice-chilled petri dish and frozen on dry ice until used to prepare raft fractions or for subcellular fractionation. Rafts were separated using a modified detergent-free method [11]. Briefly, striata from 2 rats were combined, homogenized with a Polytron and passed 20 times through a 20G needle. Following low-speed centrifugation, the supernatant was applied to a 0–25% continuous gradient of OptiPrep. After ultacentrifugation for 90 min, 12 fractions (1ml each) were collected, starting at the top of the gradient. Fractions 4–7, which contained characteristic lipid raft markers, were pooled for subsequent immunoblot or proteomic analysis.

The subcellular distribution of endogenous Rab10 in rat striatum was determined by sequential centrifugation of tissue homogenates using a published protocol [12]. The subcellular compartments analyzed included synaptosomal membranes, synaptic vesicles, a light-membrane fraction (endoplasmic reticulum, Golgi apparatus and mitochondria), and cytoplasm.

This study was carried out in strict accordance with the recommendations in the Guide for the Care and Use of Laboratory Animals of the National Institutes of Health. The protocol was approved by the Subcommittee on Animal Studies at the VA Portland Health Care System (Protocol Number 0810).

### Radioligand Binding

The 12 fractions collected from the OptiPrep gradient were analyzed for relative levels of D_1_ and D_2_ dopamine receptors by incubation with [^3^H]CH23390 (3 nM) and [^3^H]YM-09251-2 (0.5 nM), respectively, for 1 h at 25°C. Nonspecific binding was assessed using (+)-butaclamol (5 μM). Membranes were then harvested by filtration and radioactivity was measured as described previously [13].

### Proteomic Analysis

Enriched neostriatal raft proteins were run briefly into a polyacrylamide gel (50 μg/lane). The gel was cut into 6 equally spaced pieces to reduce sample complexity, and subjected to in-gel trypsin digestion. Tryptic peptides were separated by HPLC (reversed phase chromatography, C18 column) and mass spectra were obtained by electrospray ionization with a linear trap quadrupole mass spectrometer (Thermo Scientific, San Jose, CA, USA). Collision-induced dissociation and the instrument’s dynamic exclusion software were used to produce MS/MS spectra from the 3 most intense ions in each survey scan.

Peptides were identified from MS/MS scans using SEQUEST (Thermo Scientific) and an in-house processing pipeline [14] The protein database was constructed from NCBI RefSeq rat sequences (25,322 entries downloaded December 2010). The relative abundance of identified proteins between groups was measured by spectral counting. A detailed description of the proteomics methods is included in S1 File.

### Electrophoresis and Immunoblotting

Proteins were separated by sodium dodecyl sulfate-polyacrylamide gel electrophoresis under reducing conditions. The proteins were subsequently transferred to PVDF membranes and blocked with 3% bovine serum albumin in Tris-buffered saline with Tween-20 (0.05 M Tris-HCl, 0.9% NaCl, pH 7.4, 0.1% Tween-20). Blots were incubated with indicated antibodies in 0.6% bovine serum albumin overnight at 4°C. After incubation with appropriate anti-rabbit or anti-mouse horseradish peroxidase-coupled secondary antibodies, proteins were detected by use of the ECL western blotting detection kit (GE Healthcare Life Sciences, Pittsburgh, PA, USA).

### *Drosophila* Stocks

All *Drosophila* stocks were reared at 25°C using standard procedures [15]. The following fly strains were obtained from the Bloomington stock center (http://flystocks.bio.indiana.edu/): a pan-neuronal APPL-GAL4 driver (P{Appl-GAL4.G1a}1, *y* ^1^ *w* ^*^), a cholinergic Cha-GAL4 driver (P{w[+mC] = ChAT-GAL4.7.4}19B P{w[+mC] = UAS-GFP.S65T}Myo31DF[T2]) and UAS-YFP-Rab10 DN (P{UASp-YFP.Rab10.T23N}25a, y^1^ w^*^). The generation of the fly line (stock number 9786) expressing the N-terminal YFP-tagged DN form of Rab10 (T23N), under UAS control, was described previously [16]. In all experiments, the APPL-GAL4 driver line or the UAS-DN-Rab10 parental lines were used as controls.

### *Drosophila* Behavioral Assays

To quantify drug-induced activity, 2–5 day old male flies were placed in an activity monitoring system (TriKinetics). This system contains an infrared beam that passes through the midline of 65-mm long tubes, and photobeam breaks are cumulatively recorded by a computer at 10 minute bins. Methamphetamine (3 and 6 mg/ml) or caffeine (50 mM) was mixed with 2% agarose and 5% sucrose, and the activity of individual flies was monitored continuously for up to 8 days. Death was recorded at the time locomotion was no longer detected.

## Results

### Methamphetamine-Induced Changes in the Proteome of Striatal Rafts

**Fig 1:**
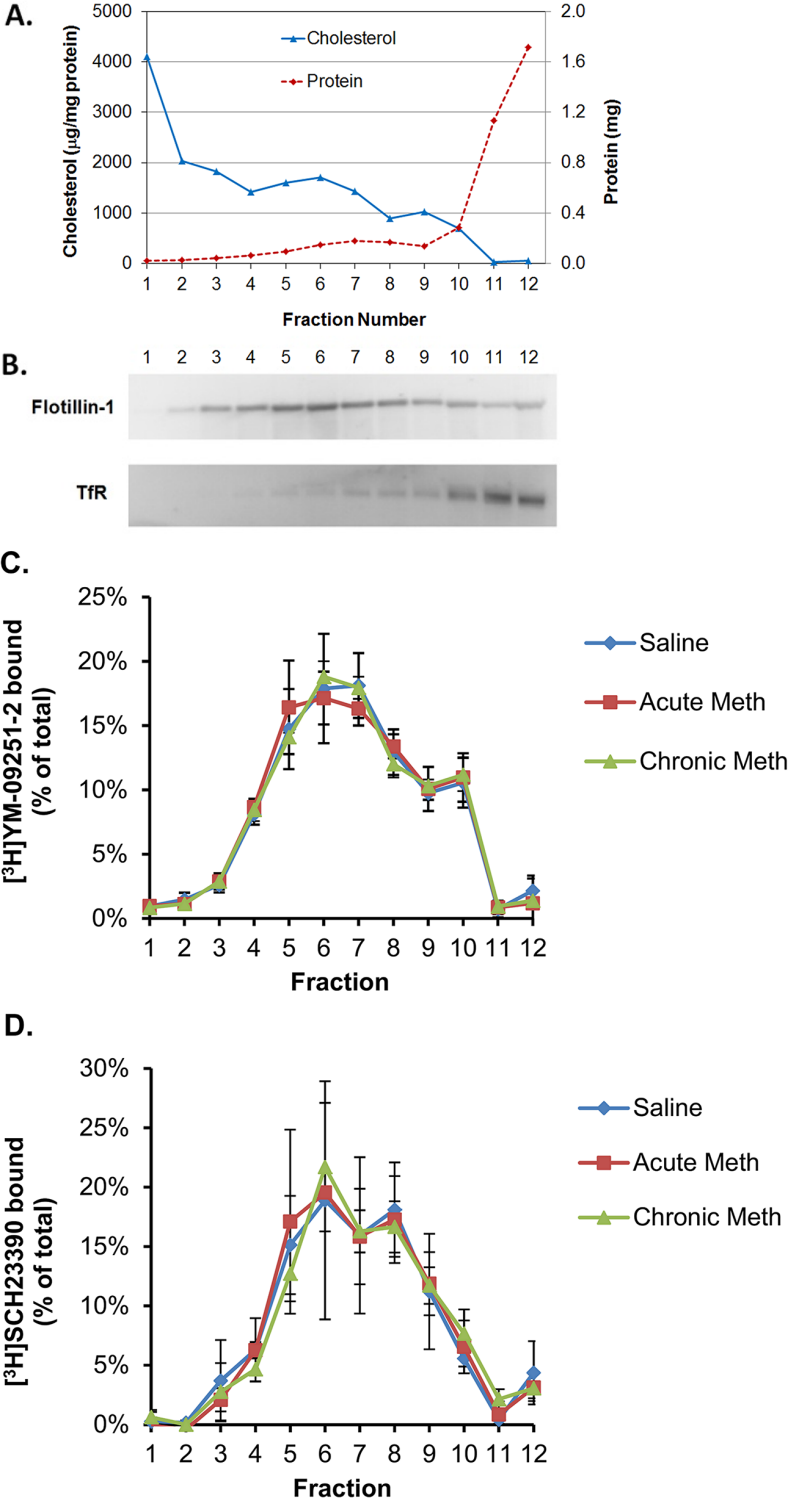
Dopamine receptors are enriched in striatal raft fractions but not affected by methamphetamine treatment. Membranes were prepared from rat striatal homogenates and separated by density gradient centrifugation. Results shown are representative of or the average of at least 3 independent experiments except where indicated. Raft fractions 4–7 were enriched in cholesterol (A), in flotillin but not transferrin receptor (TfR) or calnexin immunoreactivity (B), and in dopamine D2 (C) and D1 receptor binding activity (D; n = 2). Dopamine receptor distribution was not altered by acute or repeated treatment with methamphetamine.

Raft domains from striatal tissue (neostriatum and nucleus accumbens) were prepared from rats euthanized 30 minutes after a single injection of methamphetamine (2 mg/kg, i.p.) or after the last of 11 daily injections. Striata from 2 rats were combined for each raft preparation. The striatal raft fractions isolated by density gradient centrifugation (fractions 4–7) displayed typical raft-like characteristics, including enrichment of cholesterol and immunoreactivity for the raft marker flotillin and exclusion of the transferrin receptor and calnexin (Fig 1A and 1B). Dopamine D1 and D2 receptors were highly enriched in the raft fractions, but their relative abundance in rafts was not altered by acute or repeated treatment with methamphetamine (Fig 1C and 1D).

**Fig 2:**
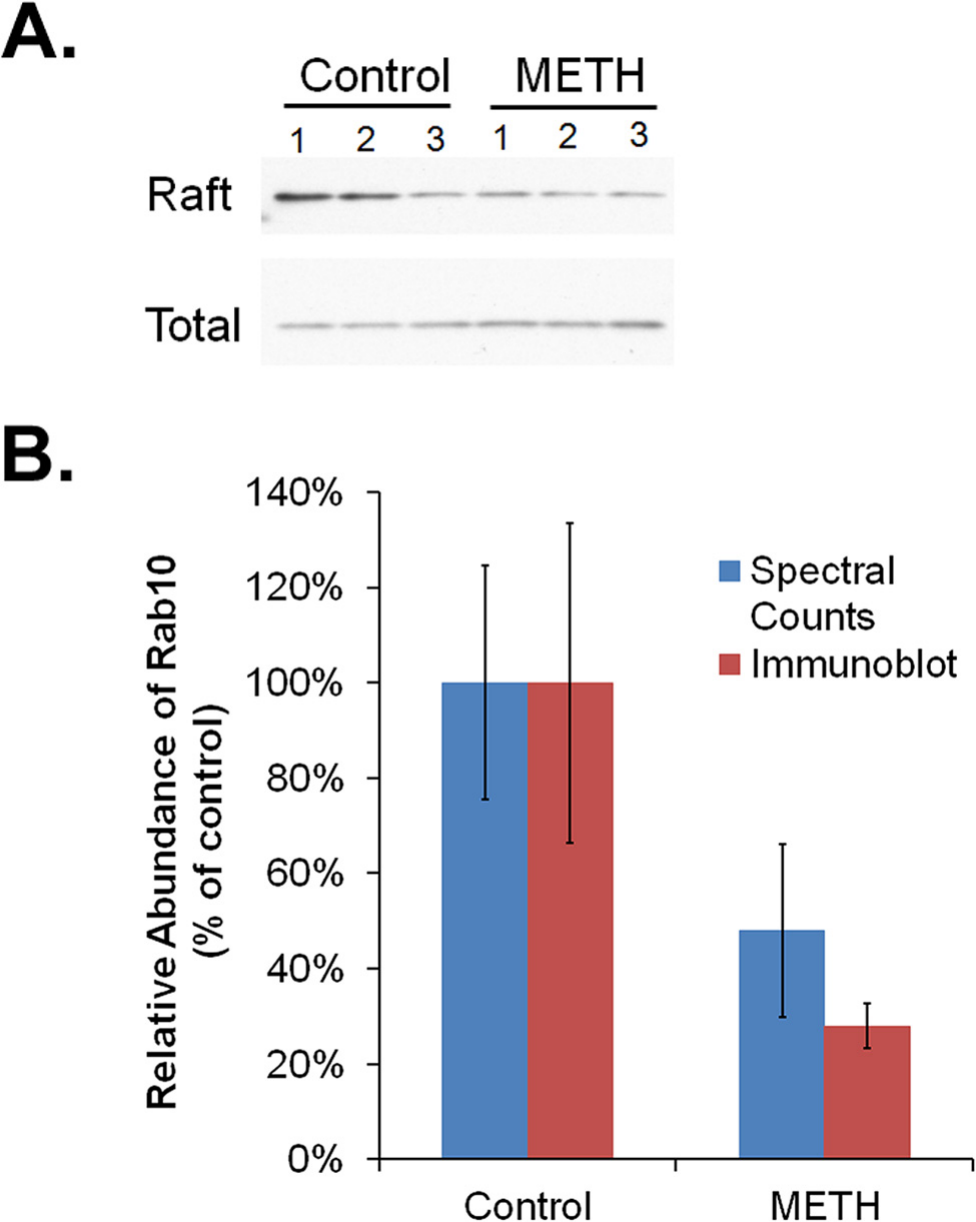
Decreased Rab10 localization in raft fractions following methamphetamine treatment. (A) Immunoblot analysis of Rab10 abundance in striatal membrane raft fractions (Raft) and total homogenates (Total) from rats treated with methamphetamine (2 mg/kg, i.p.; METH) or saline (Control), followed by density gradient centrifugation to separate raft from non-raft samples. (B) Spectral counting data for Rab10 and densitometric analysis of the immunoblots (optical density). Densitometry of Rab10 was normalized to Rab10 in total homogenates. Results shown are the mean ± SEM, expressed as a percentage of the mean control value. For spectral counting, p < 0.05 and t = 5.18 (unpaired two-tailed Student’s t-test, n = 4). For immunoblot densitometry, p = 0.10 and t = 2.92 (unpaired two-tailed Student’s t-test, n = 3).

To prepare samples for proteomic analysis, rats were treated in two independent experiments (4 rats per group per experiment) with one dose of methamphetamine or saline and raft fractions were subjected to in-gel digestion and analysis by LC-MS. In total, proteomic analysis confidently identified 1571 non-redundant proteins in raft fractions from rat striatum (excluding common contaminants and decoy sequences), including 666 proteins with high enough average spectral counts (greater than 3) across the 8 samples for reliable expression profiling and 23 proteins that displayed methamphetamine-modulated raft localization (Table 1; S1 Table; the supplementary table also includes results from rats treated with methamphetamine once daily for 11 days–“chronic”). Of these 23 proteins, 13 were significantly increased and 10 significantly decreased by acute methamphetamine treatment, compared to saline-treated rats. Rab10 was one of the proteins that decreased in abundance (2.1-fold) in rafts from rats treated acutely with methamphetamine (Table 1 and Fig 2). We decided to focus on Rab10 for additional study because of the known role of Rab10 and other monomeric G proteins in trafficking of membrane proteins via lipid rafts [17, 18].

**Table 1:** Proteins whose abundance in striatal raft fractions was altered by treatment with methamphetamine.

Proteins Altered by Acute Methamphetamine	
Protein Description	log2 (METH/Saline)
heat shock 70 kDa protein 4	1.47
potassium voltage-gated channel KCNA4	1.32
myosin-Va	1.20
myosin-9	1.19
serotransferrin precursor	1.03
Eph receptor A4	1.01
myosin-10	0.91
ser/thr-protein phosphatase 2A, PPP2R1A	0.77
PREDICTED: ankyrin 2, neuronal	0.76
striatin	0.72
microtubule-associated protein 1A	0.68
adaptor-related protein complex 2, alpha 1	0.55
clathrin heavy chain 1	0.45
NAD-dependent deacetylase sirtuin-2	-0.44
neuronal growth regulator 1 precursor	-0.50
rho-related GTP-binding protein RhoG	-0.64
opalin	-0.78
cytochrome c oxidase subunit 6B1	-0.83
ras-related protein 1A,1B group	-0.84	
probable saccharopine dehydrogenase	-1.01	
**ras-related protein Rab10**	-1.06	
ras-related protein R-Ras2	-1.19	
glycerol-3-phosphate dehydrogenase [NAD+]	-1.95	

The raft localization of 23 proteins was significantly altered by acute treatment with methamphetamine as determined by spectral counts in raft fractions from methamphetamine-treated rats verses saline-treated rats (METH/Saline). Significance thresholds were set at p < 0.05 and average spectral counts >3. Two independent experiments (n = 2 each) were combined for a total of n = 4 (4 pooled samples from 8 rats).

To confirm the altered abundance of Rab10 suggested by spectral counting, additional rats were treated with one injection of methamphetamine or saline (6 rats per group). Striata were pooled from 2 rats for isolation of rafts by density gradient centrifugation prior to assessing the abundance of Rab10 immunoreactivity by immunoblotting. Rab10 was lower in raft fractions from methamphetamine-treated rats compared to rats treated with vehicle, although the difference was not statistically significant due chiefly to the variability of the results in the control group. This was in contrast to the overall abundance of Rab10, which was unaltered by methamphetamine treatment (Fig 2).

**Fig 3:**
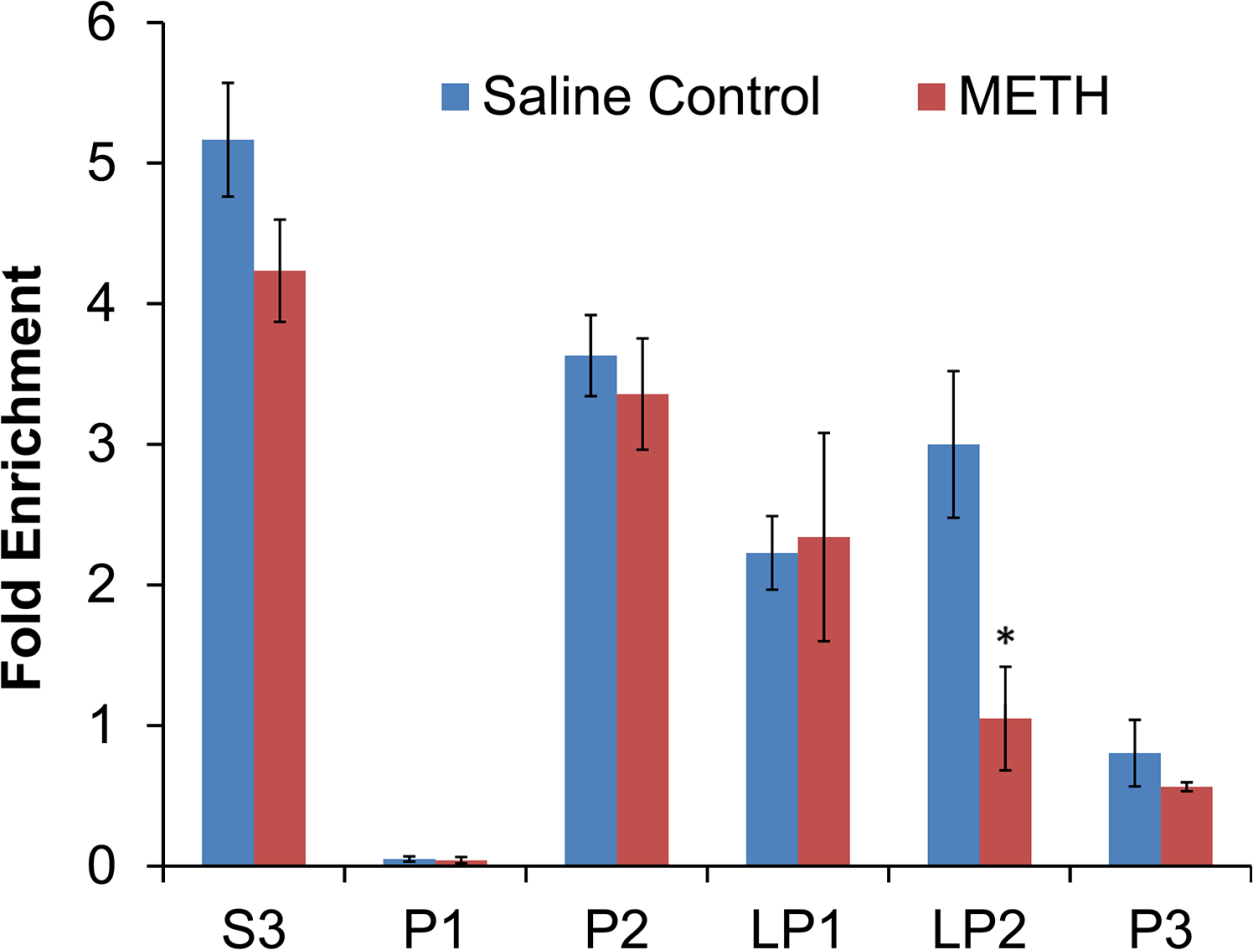
Decreased abundance of Rab10 in a vesicular membrane fraction. Rats were euthanized 30 min after one injection of methamphetamine (2 mg/kg, i.p.) or vehicle, and striatal homogenates were fractionated by differential centrifugation. S3 = cytosol, P1 = nuclei and large debris, P2 = crude synaptosomes, LP1 = synaptosome membranes, LP2 = synaptic vesicles, P3 = light membranes (non-synaptosome). The fractions and initial homogenates, containing equal amounts of protein, were loaded into SDS-PAGE gels and analyzed by western blot for Rab10 immunoreactivity. Results shown are the mean ± SEM of 4 samples, expressed as the ratio of Rab10 immunoreactivity in each fraction over Rab10 immunoreactivity in the initial homogenate. *p < 0.05 (unpaired Student’s t-test).

To identify the cellular compartment in which the abundance of Rab10 was decreased by methamphetamine, we further characterized the subcellular distribution of Rab10 in rats treated with a single dose of methamphetamine (2 mg/kg, i.p.) or saline. Subcellular compartments were purified by differential centrifugation, and immunoblot analysis revealed that, in methamphetamine-treated rats, the abundance of Rab10 was selectively decreased in a cellular compartment that is rich in synaptic vesicles (Fig 3).

### Neuronal Expression of DN-Rab10 Reduces Methamphetamine-induced Activity and Toxicity in *Drosophila melanogaster*

**Fig 4:**
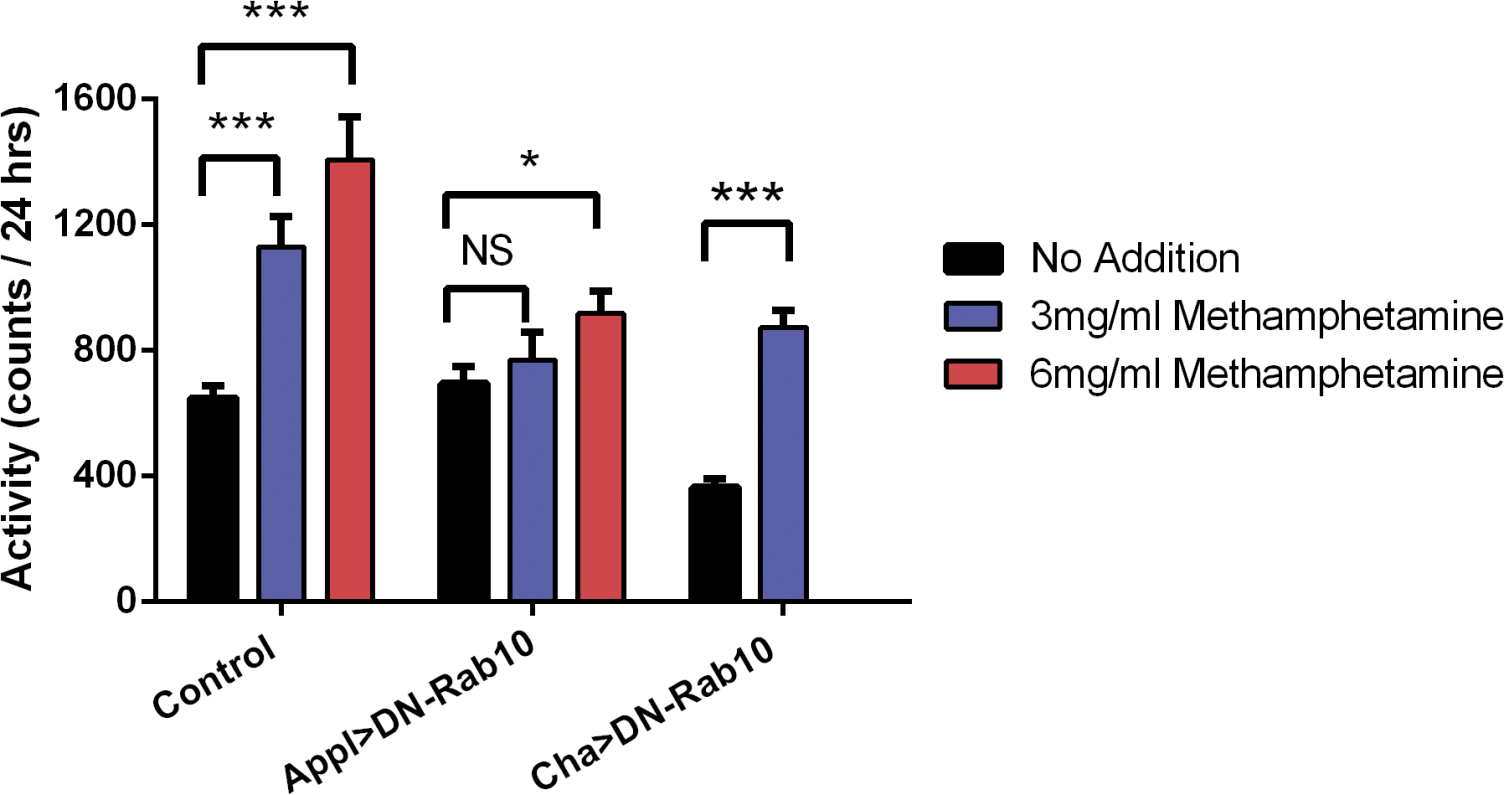
Rab10 is required for methamphetamine-induced activity in *D*. *melanogaster*. Flies were fed methamphetamine mixed into their food at the indicated concentration. The activity of the flies was continuously monitored and the total activity for the second 24 hr period tallied. Results (mean ± SEM, n = 28–30) are shown for control flies and flies expressing DN-Rab10 in all neurons (Appl>DN-Rab10) or selectively in cholinergic neurons (Cha>DN-Rab10). *p < 0.05, ***p < 0.001 compared to activity in flies fed a control diet. For pan-neuronal expression, two-way ANOVA: F (2, 172) = 16.68, p < 0.0001 for methamphetamine dose, F (1, 172) = 14.88, p = 0.0002 for genotype. Holm-Šídák multiple t-test: t-ratio (df) = 4.67 (58), 0.72 (58) and 8.35 (30) for comparing control, Appl>DN-Rab10 and Cha>DN-Rab10 genotypes respectively at 3 mg/ml and 5.54 (56) and 2.47 (58) for control and Appl>DN-Rab10 genotypes at 6mg/ml.

We used *Drosophila melanogaster* to assess the physiological significance of methamphetamine-induced changes in the localization of Rab10 because of the ease of manipulating gene expression and because of prior work evaluating the role of the raft protein flotillin in amphetamine-induced activity in fruit flies [19]. We found that behavioral activity in control flies was significantly increased when their food contained 3 or 6 mg/ml methamphetamine (Fig 4). Male flies were used for all of our experiments because we noted a slightly greater fold-change in activity, compared to female flies (S1 Fig). To test our hypothesis that methamphetamine-induced activity required the activation of Rab10, DN-Rab10, harboring a mutation in the GTP-binding domain (T23N) of *Drosophila* Rab10, was expressed in all neurons of flies using the Gal4/UAS system and the pan-neuronal Appl-GAL4 driver. Whereas the activity of control flies showed a significant increase in activity with both 3 and 6 mg/ml methamphetamine, the activity of flies expressing DN-Rab10 in all neurons us unaffected with 3mg/ml methamphetamine and only slightly increased by methamphetamine at 6 mg/ml (Fig 4). By contrast, when DN-Rab10 was expressed selectively in cholinergic neurons (sensory and many interneurons), methamphetamine (3 mg/ml) robustly increased activity compared to flies fed a control diet.

**Fig 5:**
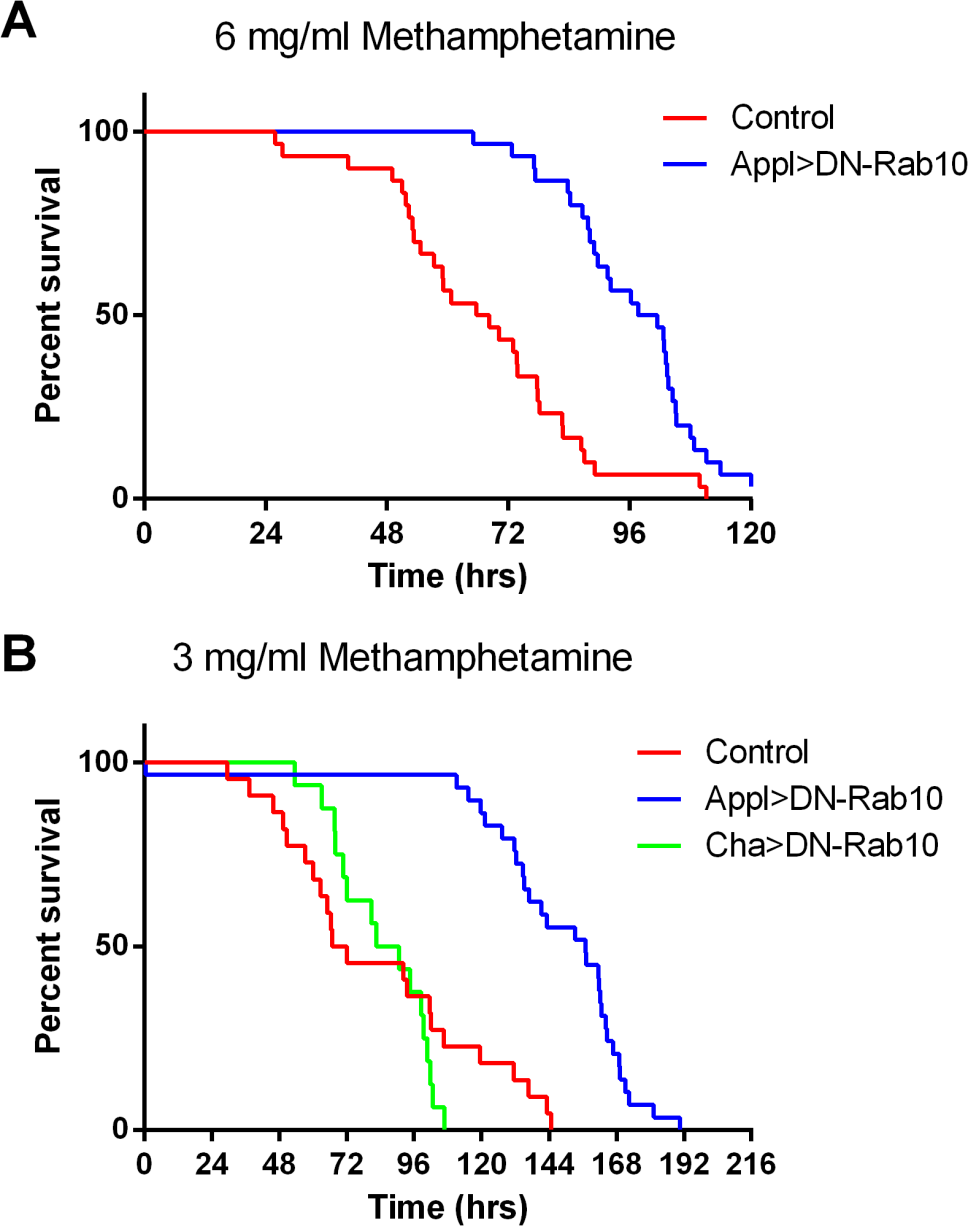
DN-Rab10 inhibits methamphetamine-induced mortality. Methamphetamine was added to the food at 3 or 6 mg/ml and the number of flies surviving over the next 8 days was determined. Death was recorded at the time activity was no longer detected. (A) The median survival time was 2.8 days for control (Appl-GAL4) flies fed 6 mg/ml methamphetamine and 4.1 days for flies expressing DN-Rab10 in all neurons (Appl>DN-Rab10). p = 0.0012 for comparison of the curves (Mantel-Cox log-rank test). The typical % survival over the same time period in flies not fed methamphetamine is greater than 90% (not shown). (B) The median survival time was 2.9 days for control flies (UAS-DN-Rab10), 6.5 days for flies expressing DN-Rab10 in all neurons (Appl>DN-Rab10) and 3.6 days for flies expressing DN-Rab10 in cholinergic neurons (Cha>DN-Rab10) and fed 3 mg/ml methamphetamine. P < 0.0001 for the comparison of the control and Appl>DN-Rab10 curves and p = 0.49 for the comparison of the control and Cha>DN-Rab10 curves.

We observed that mortality was markedly higher among flies fed methamphetamine (Fig 5). To determine if expression of DN-Rab10 would improve the survival of flies exposed to methamphetamine, we counted the number of surviving control flies and those expressing DN-Rab10 in all neurons over the course of 5 days (Fig 5A). DN-Rab10 expression in all neurons resulted in a significant increase in survival in flies fed 6 mg/ml methamphetamine. The median survival was 2.8 days for the control flies and 4.1 days for the Appl>DN-Rab10 flies. In a second experiment we determined that pan-neuronal expression of DN-Rab10, but not expression specifically in cholinergic neurons, also protected against lethality in flies fed methamphetamine at a concentration of 3 mg/ml (Fig 5B)

To verify that the observed changes in both methamphetamine-induced activity and survival were not due to lower ingestion of methamphetamine by flies expressing DN-Rab10, we measured methamphetamine concentrations by ELISA in wild-type and DN-Rab10 flies receiving 3 or 6 mg/ml methamphetamine for 24 hr. We found that there was no significant difference in the quantity of methamphetamine ingested between the two groups of flies. The methamphetamine concentrations after 24 hr were 48 ± 12 (mean ± SEM) and 150 ± 18 ng/fly in control flies given 3 and 6 mg/ml methamphetamine, respectively, and 45 ± 19 and 107 ± 29 in DN-Rab10 flies receiving 3 mg/ml and 6 mg/ml methamphetamine, respectively (n = 6–7 groups of 7–14 flies).

**Fig 6:**
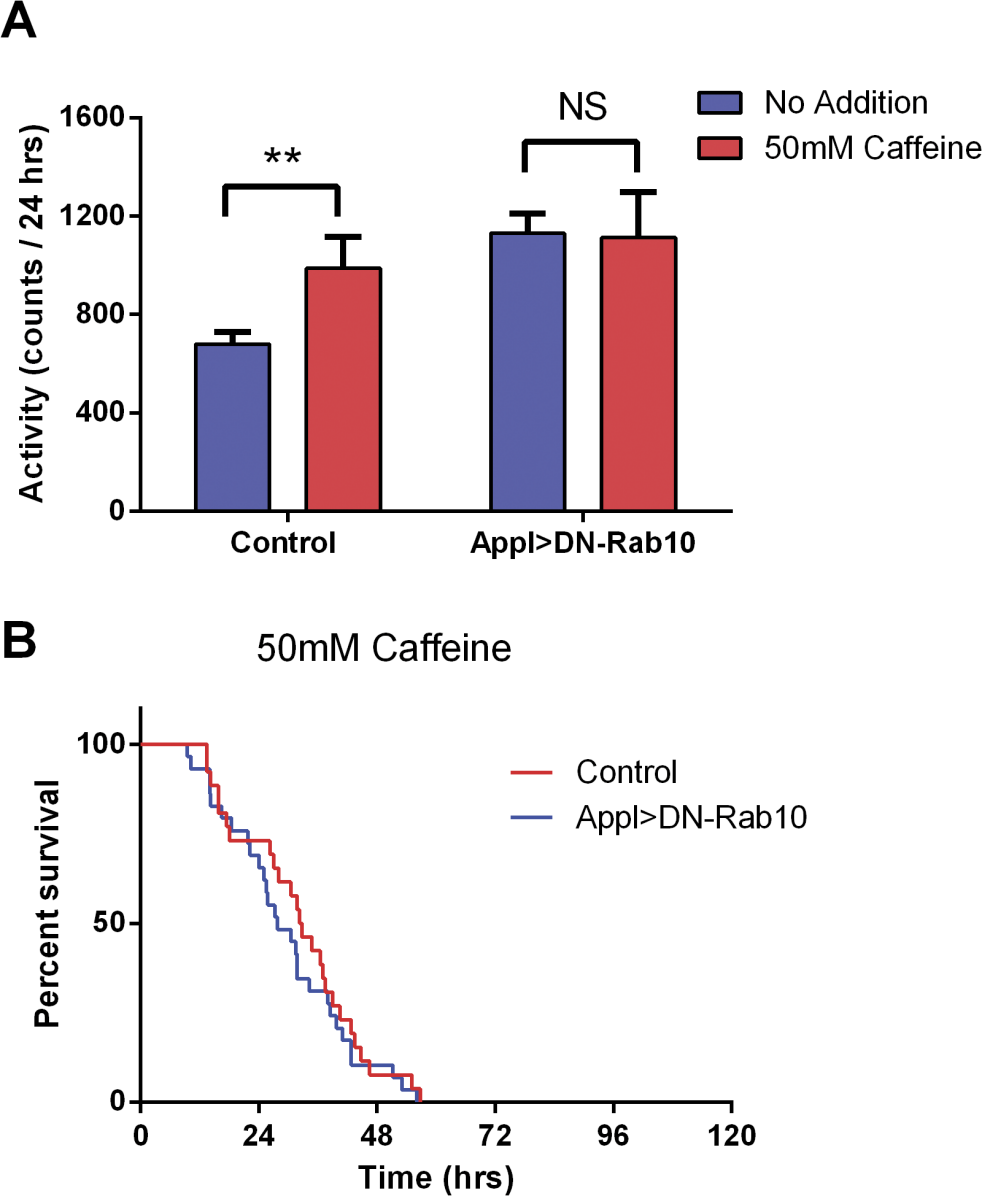
DN-Rab10 inhibits caffeine induced increased activity but not mortality. (A) Activity. Flies were fed food containing 50 mM caffeine and their activity monitored with the same procedure as methamphetamine treatment. Control flies showed an increased activity in the presence of caffeine, whereas flies expressing DN-Rab10 in all neurons showed no increase. Two-way ANOVA: F (1, 74) = 7.32, p < 0.01 for caffeine and F (1,74) = 1.87, p > 0.058 for genotype. Sidak's multiple comparisons test: *p < 0.05, t = 2.309 for control flies and p > 0.05, t = 0.103 for flies expressing DN-Rab10 in all neurons. (B) Mortality. The mean survival time was 33 hours for control flies and 28 hours for flies expressing DN-Rab10 in all neurons (Appl>DN-Rab10).

To assess the generalizability of the role of Rab10 10 in behavioral responses to psychostimulants, flies were fed a diet containing 50 mM caffeine and activity was measured for the next 24 hr. Caffeine significantly increased activity in control flies, but not in flies with pan-neuronal expression of DN-Rab10 (Fig 6A), although in this experiment the activity of flies fed a control diet was enhanced by expression of DN-Rab10. Qualitatively similar results were observed in the second day after addition of caffeine to the food (data not shown), but this concentration of caffeine resulted in significant mortality during that second day. Caffeine-induced mortality was not altered by pan-neuronal expression of DN-Rab10 (Fig 6B).

## Discussion

In this study we demonstrated that acute or repeated treatment with methamphetamine did not alter the distribution of dopamine D1 and D2 receptors between raft and non-raft membrane fractions in rat neostriatal membranes. A proteomic analysis, however, indicated that the relative abundance in rafts of a number of other proteins was modulated by methamphetamine, including the monomeric GTP-binding protein Rab10. Inhibiting the activity of neuronal Rab10 with DN-Rab10 in *D*. *melanogaster* also inhibited methamphetamine-induced activity and mortality.

Lipid rafts contribute importantly to the function of the dopamine system. D1 and D2 receptors are both concentrated in detergent-resistant membrane fractions under some conditions [20,21], although perhaps in a detergent-resistant fraction distinct from flotillin-rich rafts in human embryonic kidney 293 cells [22]. Dopamine D1 receptors are concentrated in a subset of lipid rafts called caveolae that contain caveolin, and D1 receptor interaction with caveolin regulates D1 receptor signaling and internalization [23,24]. Disrupting lipid rafts increases the mobility of the dopamine transporter in the plasma membrane of living cells [25]. The raft protein flotillin is required for localization of the dopamine transporter in lipid rafts, and also for protein kinase C-regulated internalization of the dopamine transporter and amphetamine-induced dopamine release [19,26]. Our results confirm that rat striatal D1 and D2 receptors are enriched in fractions that also contain flotillin. Agonist-induced D1 receptor interaction with caveolins and translocation into caveolin-rich fractions has been reported [23,24], and daily treatment with cocaine causes translocation of D1 receptor immunoreactivity, but not D2 receptor immunoreactivity, from detergent-resistant (i.e., raft) fractions to detergent-soluble fractions of the rat frontal cortex [20], we found that treating rats with methamphetamine, which would be expected to activate both D1 and D2 dopamine receptors because of enhanced dopamine release, produced no detectable change in the distribution of striatal dopamine receptors.

A proteomic analysis of rat striatal raft fractions identified 23 proteins whose abundance in raft fractions was altered by a single administration of methamphetamine. One of the proteins whose abundance in raft fractions was decreased by methamphetamine was Rab10. Rab GTPases are a family of proteins that cycle between an inactive GDP-bound state and an active GTP-bound state. They are anchored to membranes via prenylation at their C-termini and, in the GTP-bound state, recruit effector proteins to control vesicular transport between organelles by mechanisms involving cargo selection, vesicle budding, vesicle fusion, and the transport of vesicles along the cytoskeleton [17]. Rab10 contributes to the trafficking of newly synthesized proteins from the *trans*-Golgi network to the plasma membrane or the recycling of endocytosed membrane proteins [27–33], with perhaps the best-characterized role for Rab10 being its requisite function for insulin-induced translocation of the glucose transporter GLUT4 to the cell surface in adipocytes and muscle cells [34–36]. As G protein-coupled receptors are frequently found in raft domains, it is notable that a proteomic study identified Rab10 as an interactor with the MT1 melatonin receptor [18]. The location of Rab10 in rafts is also supported by the finding that it functions in a cholesterol-dependent pathway for the recycling of glutamate receptors in *Caenorhabditis elegans* [27]. Moreover, the related GTPase H-ras resides in rafts in the GDP-complexed state, and moves into non-raft membranes upon GTP loading, where it is biologically active [37].

Recognizing that altered localization of Rab10 could reflect methamphetamine-induced alteration of the activation state of this protein known to be important for trafficking of synaptic proteins, we focused on Rab10 for subsequent experiments. We used a separate group of rats to provide tentative support for the finding that methamphetamine treatment decreased the localization of Rab10 in raft fractions without affecting overall abundance, and also determined by subcellular fractionation that methamphetamine treatment reduced the abundance of Rab10 in a membrane fraction that includes synaptic vesicles and endosomes. That vesicular fraction contains a very small percentage of the total protein (~0.1%), so the decrease in that compartment was not accompanied by a detectable increase in other compartments that contained much higher percentages of the total protein. The observed localization of Rab10 in synaptic vesicles is in agreement with a previous report [38].

We turned to *Drosophila melanogaster*, a highly tractable genetic model organism that has often been used to study the effects of psychostimulants and other abused drugs [26,39,40], to assess a potential role for activated Rab10 in methamphetamine-induced behavior. Fruit flies express orthologs of Rab10 [41] and most mammalian proteins involved in dopamine signaling, including tyrosine hydroxylase expressed specifically in dopamine neurons [42,43], the dopamine transporter [44], the vesicular monoamine transporter [45], and both D1-like dopamine receptors that stimulate adenylate cyclase [46,47] and a D2-like dopamine receptor that is inhibitory and is expressed in both non-dopaminergic neurons and in dopamine neurons where it behaves as an autoreceptor [48,49]. We determined that methamphetamine, mixed in the food made available to the flies, increased the activity and decreased the survival of the flies. Furthermore, pan-neuronal expression of a dominant negative mutant of Rab10 in which threonine-23 in the GTP-binding domain was changed to asparagine [50] inhibited methamphetamine-induced activity and mortality caused by methamphetamine, indicating that Rab10 is required for full behavioral responsiveness to methamphetamine. Expression of DN-Rab10 specifically in cholinergic neurons had no effect on methamphetamine effects on activity or mortality. Interestingly, DN-Rab10 also blocked caffeine-induced activity, whereas it had no effect on mortality, suggesting that these consequences of Rab10 activity are due to two separable mechanisms. For example, one could speculate that caffeine-stimulated activity, as well as methamphetamine-stimulated activity and mortality, is mediated by stimulation of dopamine release or enhanced dopamine receptor-mediated signaling [51], whereas effects of caffeine on mortality involve other brain or peripheral mechanisms. We propose that methamphetamine-induced redistribution of Rab10 reflects either the involvement of Rab10 in methamphetamine-induced dopamine release or downstream activation of dopamine receptor-mediated signaling.

## Supporting Information

S1 Fig Methamphetamine Response in Male and Female Flies. Wild type flies were fed either methamphetamine mixed into their food at 6 mg/ml or normal diet (control). The panels on the left show activity profiles for 5 days. The panels on the right show average activity counts/24 hr over the first 48 hr of the experiment. Results shown are mean ± SEM. n = 16.
(TIF)

S1 File Detailed Proteomic Methods. (DOC)

S1 Table Detailed Proteomic Results. (XLS)
