# Supplementary figures and images for: Role for Rab10 in Methamphetamine-Induced Behavior
Source: PLoS One. 2015 Aug 20;10(8):e0136167. doi: 10.1371/journal.pone.0136167 (PMC4546301; doi:10.1371/journal.pone.0136167)

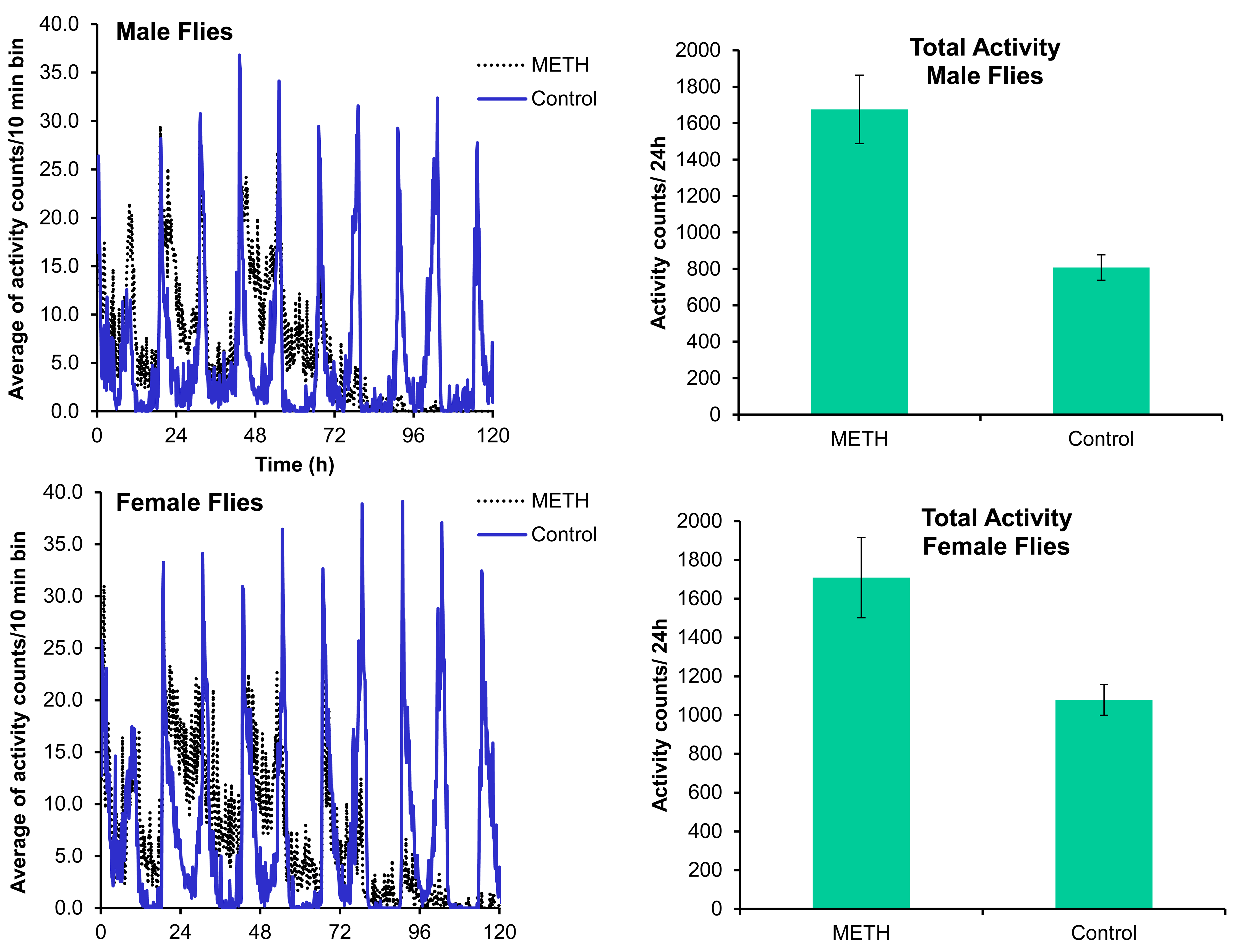

Supplement: S1 Fig — Wild type flies were fed either methamphetamine mixed into their food at 6 mg/ml or normal diet (control). The panels on the left show activity profiles for 5 days. The panels on the right show average activity counts/24 hr over the first 48 hr of the experiment. Results shown are mean ± SEM. n = 16. (TIF) [file pone.0136167.s001.tif]
