# Supplementary material for: Role for Rab10 in Methamphetamine-Induced Behavior
Source: PLoS One. 2015 Aug 20;10(8):e0136167. doi: 10.1371/journal.pone.0136167 (PMC4546301; doi:10.1371/journal.pone.0136167)
Supplement: S1 File — (DOC) [file pone.0136167.s002.doc]

Supplementary file describing proteomics methods

Role for Rab10 in Methamphetamine-Induced Behavior

Scott M. Vanderwerf, David C. Buck, Philip A. Wilmarth, Leila M. Sears, Larry L. David, David B. Morton, and Kim A. Neve

A 50 μg portion of rat lipid raft preparation was applied to a single lane of a 10% Novex NuPage Bis Tris gel, run at 200V in MOPS buffer for 30 min, the gel stained with Imperial Coomassie (Pierce Chemical), the gel imaged with a flat bed scanner, placed on a clean glass plate, and the lane cut into 6 equally spaced regions from the top of the lane to the bottom of the tracking dye. The excised gel slices were then cut into 1-2 mm sections with a razor blade, placed into a 0.5 ml centrifuge tube, washed twice by shaking in 0.5 ml of water for 15 min, washed twice by shaking in 0.5 ml of 1:1 solution of acetotnitrile/100 mM ammonium bicarbonate for 30 min, then dried by vacuum centrifugation. Following reduction and alkylation of proteins in the excised gel slices by incubation first in DTT then in iodoacetamide, proteins were digested by rehydrating the gel slice on ice in trypsin digestion solution containing 100 mM ammonium bicarbonate and 10 μg/ml proteomics grade modified trypsin (Sigma). The excess trypsin solution was then removed, and the gel pieces covered with 100 mM ammonium bicarbonate solution and the tube incubated overnight at 37°C. The solution was then removed from the gel slices, additional peptides extracted, the combined volume decreased by vacuum centrifugation and peptides transferred directly to auto-sampler tubes for analysis.

Isolated peptides were then injected onto a 1 mm x 8 mm peptide trap cartridge (Optimize Technologies, Inc.) at 20 μl/minute in a mobile phase containing 0.1% formic acid. The trap cartridge was then placed in-line with a 0.5 mm x 250 mm column containing 5 μm Zorbax SB-C18 stationary phase (Agilent Technologies), and peptides separated by a 2-30% acetonitrile gradient over 195 minutes at 10 μl/minute using a 1100 series capillary HPLC (Agilent Technologies). Peptides were analyzed using a LTQ linear ion trap fitted with an Ion Max Source and 34-gauge metal needle kit (Thermo Scientific). Survey mass spectrometry (MS) scans from m/z 400-2000 were alternated with 3 data-dependant MS/MS scans using the dynamic exclusion feature of the software to increase the number of unique peptides analyzed (repeat count equal to 1, exclusion list size of 50, exclusion duration of 30 sec, and exclusion mass width of -1 to +1.5). The tune file was configured with no averaging of microscans, a maximum inject time of 200 msec for both MS and MSn scans, and AGC targets of 3 x 10^4^ in MS mode and 1 x 10^4^ in MSn mode.

Peptide identification was performed by comparing observed MS/MS spectra to theoretical fragmentation spectra of peptides generated from a protein database. A rat species subset of NCBI RefSeq protein database (25,322 sequences downloaded Dec. 9, 2010) was prepared with concatenated sequence-reversed entries (and common contaminants) and searched with SEQUEST [1]. A parent ion mass tolerance of 2.5 Da was used with average parent ion mass and monoisotopic fragment ion masses. Cysteine had a static modification mass of +57 Da; methionine had a variable modification mass of +16 Da; and serine, threonine, and tyrosine had variable modification masses of +80 Da. An in-house suite of programs [2] were used to provide a Peptide Prophet-like [3] discriminant function scoring to identify “correct” peptides and discard “incorrect” peptides using sequence-reversed matches to estimate false discovery rates [4]. Protein identification lists were prepared using basic parsimony logic [5] with post processing of results to improve spectral count accuracy and allow more strict protein identification criteria. Proteins were considered present in a given sample if they had two or more fully-tryptic peptides with distinct sequences.

Protein expression levels between pairs of treatment sample sets were estimated using spectral counting [6] with quantile normalization [7] to account for run-to-run instrument variation. We used a minimum average spectral count threshold of 3.0 to remove proteins with low, variable counts and proteins with many missed observations across the samples. Proteins having large fractions of peptides shared with other proteins (e.g. common house keeping proteins such as tubulins and actins) were combined into groups before quantitative comparisons. We used a standard unpaired two-sample, two-tailed student’s t-test to compute expression difference for the 666 proteins/protein groups passing the minimum count cutoff. We also used a sliding window (51 proteins wide running from highest spectral count to lowest) Z-transformation [8] of the average expression ratios. Z-score p-values were computed based on Gaussian fits of the Z-score distributions. We did not apply any multiple testing corrections of p-values in this discovery phase. However, a combination of significance in each test was used to determine more confident final differential expression candidates.

**References:**

1. Eng, Jimmy K., Ashley L. McCormack, and John R. Yates. "An approach to correlate tandem mass spectral data of peptides with amino acid sequences in a protein database." *Journal of the American Society for Mass Spectrometry* 5.11 (1994): 976-989.

2. Wilmarth, Phillip A., Michael A. Riviere, and Larry L. David. "Techniques for accurate protein identification in shotgun proteomic studies of human, mouse, bovine, and chicken lenses." *Journal of ocular biology, diseases, and informatics* 2.4 (2009): 223-234.

3. Keller, Andrew, Alexey I. Nesvizhskii, Eugene Kolker, and Ruedi Aebersold. "Empirical statistical model to estimate the accuracy of peptide identifications made by MS/MS and database search." *Analytical chemistry* 74, no. 20 (2002): 5383-5392.

4. Elias, Joshua E., and Steven P. Gygi. "Target-decoy search strategy for increased confidence in large-scale protein identifications by mass spectrometry." *Nature methods* 4.3 (2007): 207-214.

5. Nesvizhskii, Alexey I., and Ruedi Aebersold. "Interpretation of shotgun proteomic data the protein inference problem." *Molecular & Cellular Proteomics* 4.10 (2005): 1419-1440.

6. Liu, Hongbin, Rovshan G. Sadygov, and John R. Yates. "A model for random sampling and estimation of relative protein abundance in shotgun proteomics." *Analytical chemistry* 76.14 (2004): 4193-4201.

7. Bolstad, Benjamin M., Rafael A. Irizarry, Magnus Åstrand, and Terence P. Speed. "A comparison of normalization methods for high density oligonucleotide array data based on variance and bias." *Bioinformatics* 19, no. 2 (2003): 185-193.

8.Yang, Ivana V., Emily Chen, Jeremy P. Hasseman, Wei Liang, Bryan C. Frank, Shuibang Wang, Vasily Sharov et al. "Within the fold: assessing differential expression measures and reproducibility in microarray assays." *Genome Biol* 3, no. 11 (2002): 1-0062.
